# Zilebesiran, a Small Interfering RNA Therapeutic Targeting Angiotensinogen: Mechanism, Clinical Evidence, Safety, and Implementation Considerations

**DOI:** 10.3390/life16081301

**Published:** 2026-08-08

**Authors:** Jawaria, Areeba Noor, Yusra Zarlashat, Muhammad Ebad Asif Khan, Enrique Mandado Loureiro, Edit Dósa

**Affiliations:** 1 Health Biotechnology Division, National Institute for Biotechnology and Genetic Engineering-C (NIBGE), Faisalabad 38000, Pakistan; jawariasiddiqui100@gmail.com; 2 Pakistan Institute of Engineering and Applied Sciences (PIEAS), Islamabad 45650, Pakistan; 3 Department of Bioinformatics and Biotechnology, Government College University Faisalabad, Faisalabad 38000, Pakistan; areeba.202204164@gcuf.edu.pk; 4 Department of Biochemistry, Government College University Faisalabad, Faisalabad 38000, Pakistan; yusrazarlashat@gcuf.edu.pk; 5 Heart and Vascular Center, Semmelweis University, 1122 Budapest, Hungary; muhammad.khan@stud.semmelweis.hu (M.E.A.K.); enrique.mandado@stud.semmelweis.hu (E.M.L.)

**Keywords:** zilebesiran, hypertension, angiotensinogen, small interfering RNA, RNA interference, GalNAc-conjugated siRNA, renin–angiotensin–aldosterone system, precision medicine

## Abstract

Hypertension remains a major global health burden, and control rates remain suboptimal because of poor medication adherence and limitations of existing therapies, including escape within the renin–angiotensin–aldosterone system. Zilebesiran, a first-in-class, subcutaneously administered small interfering RNA therapeutic, represents a promising advance in hypertension management. Through N-acetylgalactosamine-mediated hepatic delivery, zilebesiran selectively silences angiotensinogen (AGT) messenger RNA, the transcript encoding the common precursor of all angiotensin peptides. This upstream intervention reduces AGT production and produces durable blood pressure lowering that can persist for up to 6 months after a single dose. This review summarizes the mechanism of action of zilebesiran, its pharmacokinetic and pharmacodynamic properties, and the available phase 1 and phase 2 clinical evidence, including the KARDIA program. We also place zilebesiran within the evolving antihypertensive landscape by comparing it with aldosterone synthase inhibitors, dual endothelin receptor antagonists, and brain aminopeptidase A inhibitors. Finally, we discuss translational challenges, including reversal strategies for emergency situations, monitoring considerations, and potential roles for personalized dosing. Early-phase and phase 2 trials show dose-dependent and durable reductions in serum AGT and blood pressure with infrequent dosing; however, long-term safety, cardiovascular outcome benefit, and generalizability in diverse high-risk populations remain to be established, and zilebesiran remains investigational while phase 3 outcome testing is underway.

## 1. Introduction

Hypertension remains a leading contributor to cardiovascular morbidity and mortality worldwide [1,2]. More than 1.3 billion adults are affected globally [3], and prevalence has approximately doubled over the past three decades, with further increases expected [4]. Despite the availability of several antihypertensive drug classes, blood pressure control remains inadequate, and fewer than half of treated patients achieve target levels [5]. Poor adherence to daily therapy, driven in part by pill burden, is a major reason for this gap [6,7]. In addition, the efficacy of angiotensin-converting enzyme (ACE) inhibitors and angiotensin receptor blockers (ARBs) may be attenuated over time by renin–angiotensin–aldosterone system (RAAS) escape, a compensatory response characterized by increased renin secretion and renewed angiotensin II (Ang II) generation [8].

The RAAS is a central regulator of blood pressure, fluid balance, vascular tone, and aldosterone synthesis [9]. Ang II increases blood pressure through vasoconstriction, renal sodium retention, aldosterone release, and sympathetic activation. Accordingly, suppression of hepatic angiotensinogen (AGT) offers an attractive therapeutic strategy for hypertension. Preclinical and clinical studies suggest that AGT-directed small interfering RNA (siRNA) can produce sustained blood pressure reduction [10,11,12,13]. This mechanism may overcome some limitations of conventional RAAS inhibitors, including incomplete pathway suppression and RAAS escape [14,15].

## 2. Scope and Search Strategy

Recent reviews have summarized RNA-based antihypertensive therapies and zilebesiran more broadly [16,17]. In contrast, this review emphasizes the clinical and translational implications of zilebesiran, including monitoring, high-risk populations, reversal strategies, and its positioning relative to other emerging antihypertensive classes. This narrative review was informed by searches of PubMed/MEDLINE and Scopus from January 2017 through May 2026 using combinations of the terms “zilebesiran”, “angiotensinogen”, “hypertension”, “small interfering RNA”, and “N-acetylgalactosamine (GalNAc)-conjugated siRNA”. Reference lists of eligible articles and relevant reviews were also screened. To capture late-breaking but clinically relevant updates, we additionally reviewed ClinicalTrials.gov records, major cardiology congress presentations related to KARDIA-3 and ZENITH, and regulatory materials relevant to aprocitentan and baxdrostat. Priority was given to peer-reviewed clinical trials and high-quality reviews; preclinical mechanistic and pharmacokinetic/pharmacodynamic studies were included to provide biological context. Because this was a focused narrative review rather than a formal systematic review, no prespecified protocol or formal risk-of-bias tool was applied. Evidence derived from conference presentations, company reports, or trial registries was treated as non-peer-reviewed and is explicitly identified as such in the text and tables; these sources were used for recent trial results or status information when a full peer-reviewed report was unavailable.

## 3. Emerging siRNA Therapeutics for Targeting Hepatic AGT

RNA interference (RNAi) is an evolutionarily conserved gene-silencing mechanism that has been translated into clinically useful siRNA therapeutics [18,19]. This platform enables selective suppression of disease-relevant gene expression and has become a major advance in nucleic acid therapeutics [20]. Within this field, zilebesiran is a leading candidate for hypertension management. Its GalNAc conjugate binds the hepatic asialoglycoprotein receptor (ASGPR; Ashwell–Morell receptor), facilitating hepatocyte uptake and subsequent suppression of AGT messenger RNA (mRNA) [11]. Because hepatic AGT is the source of all downstream angiotensin peptides, this approach lowers angiotensin I and Ang II and can produce clinically meaningful blood pressure reduction [21]. Conventional RAAS inhibitors, by contrast, can be limited by compensatory activation and RAAS escape [14,15,22]. By reducing AGT availability upstream of renin activity, RNAi-mediated AGT inhibition may mitigate this problem while retaining the benefits of targeted hepatic delivery and limited off-target exposure [8,22].

Several siRNA therapeutics have now received regulatory approval, marking important milestones for the field. Prominent examples include inclisiran, lumasiran, givosiran, and patisiran [23], each developed for a distinct pathophysiologic target ranging from hypercholesterolemia to hereditary transthyretin amyloidosis [24,25]. Within this expanding therapeutic class, zilebesiran stands out as a promising candidate for hypertension because it targets hepatic AGT after subcutaneous administration and offers the prospect of durable blood pressure control with infrequent dosing [26].

## 4. Mechanism of Zilebesiran and Its Distinctive Biology

GalNAc-conjugated siRNA is a state-of-the-art platform for hepatocyte-selective suppression of target mRNA [27]. Zilebesiran consists of a chemically modified double-stranded siRNA duplex, comprising guide and passenger strands, linked to a trivalent GalNAc ligand that binds the ASGPR on hepatocytes. Contemporary GalNAc–siRNA constructs typically incorporate extensive 2′-O-methyl and 2′-fluoro ribose substitutions with strategically placed phosphorothioate linkages to improve nuclease resistance and intracellular persistence; the complete sequence-specific modification pattern of zilebesiran has not been detailed in peer-reviewed clinical reports [28,29]. Following endocytosis and endosomal escape, the duplex is loaded onto Argonaute 2 (Ago2), the catalytic component of the RNA-induced silencing complex (RISC). Ago2 cleaves and removes the passenger strand, whose fragments may persist transiently, while the retained guide strand directs sequence-specific cleavage of AGT mRNA and reduces hepatic AGT synthesis [28,29,30,31].

**Figure 1:**
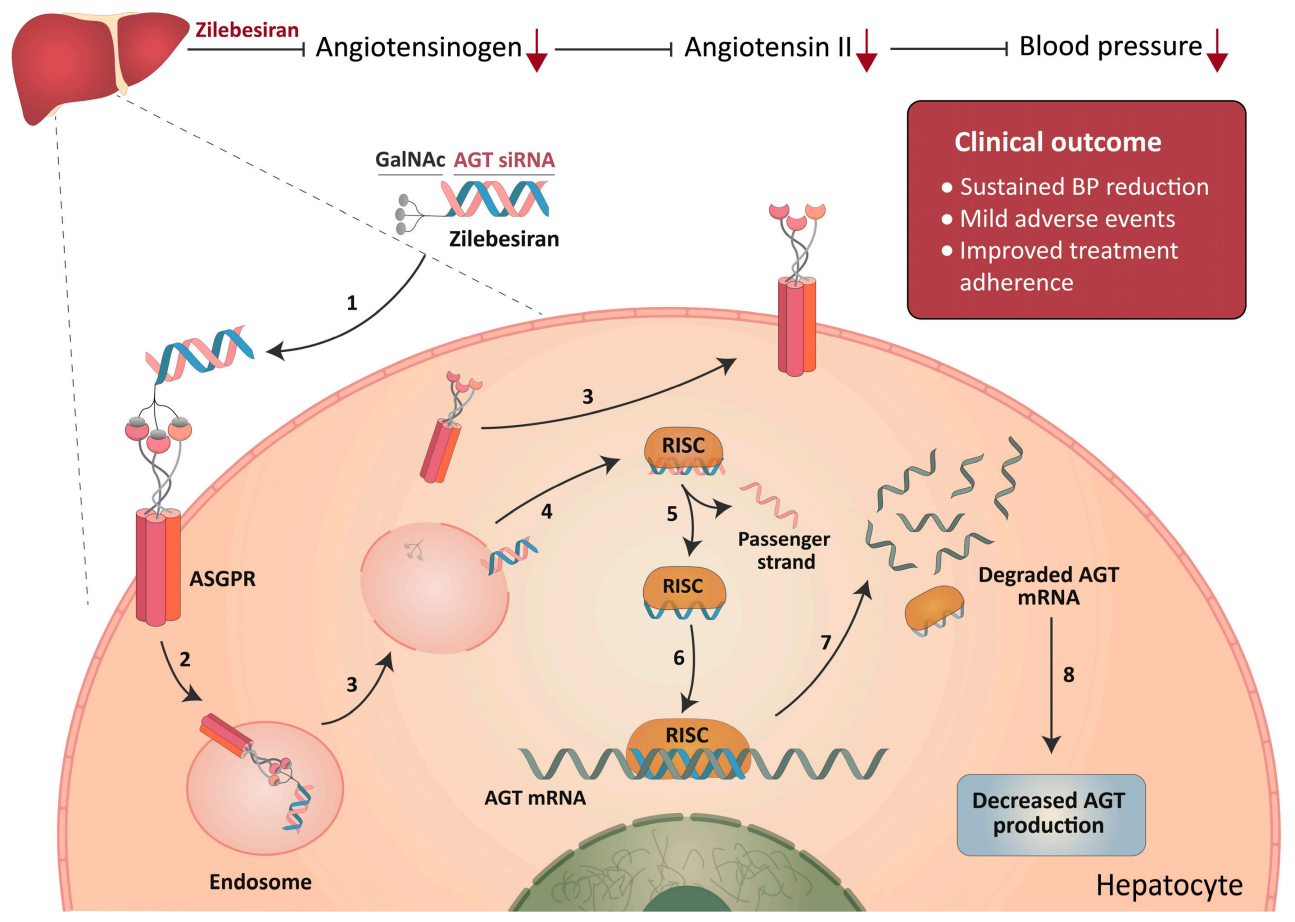
Mechanism of action of zilebesiran, a GalNAc-conjugated siRNA targeting hepatic AGT mRNA. (1) Binding to ASGPR; (2) clathrin-mediated endocytosis of the siRNA–receptor complex; (3) endosomal escape and ASGPR recycling; (4) loading of the duplex onto Ago2 to form the catalytic RISC; (5) Ago2-mediated passenger-strand cleavage and unwinding, with transient cleaved fragments; (6) guide-loaded RISC recognition of AGT mRNA; (7) cleavage and degradation of AGT mRNA; and (8) decreased hepatic AGT production and downstream RAAS activity. Ago2—Argonaute 2; AGT—angiotensinogen; ASGPR—asialoglycoprotein receptor; BP—blood pressure; GalNAc—N-acetylgalactosamine; mRNA—messenger RNA; RAAS—renin–angiotensin–aldosterone system; RISC—RNA-induced silencing complex; siRNA—small interfering RNA.

A key biological distinction of zilebesiran is its chemically stabilized GalNAc–siRNA design, which supports prolonged pharmacodynamic effects [28,29]. Although circulating AGT is produced predominantly by the liver, local RAAS activity in the kidney, heart, vasculature, brain, and adipose tissue contributes to sodium handling, vascular and cardiac remodeling, and neurohumoral regulation [9,32,33,34]. The extent to which liver-directed AGT silencing alters these tissue systems remains uncertain and is relevant to safety during volume depletion, shock, and pregnancy. Figure 1 summarizes the mechanism of zilebesiran.

## 5. Clinical Evidence: Efficacy and Safety of Zilebesiran

Several clinical studies now provide a preliminary evidence base for the antihypertensive efficacy and short-term safety of zilebesiran.

### 5.1. Phase 1 Clinical Trial

The first clinical evaluation of zilebesiran was a phase 1 randomized, placebo-controlled study involving 107 adults with treated or untreated hypertension [13]. Part A enrolled 84 participants, randomly assigned in a 2:1 ratio to a single ascending subcutaneous dose of zilebesiran (10 to 800 mg) or placebo and followed them for 24 weeks. By week 8, dose-dependent reductions were observed in both serum AGT (r = −0.56; 95% confidence interval, −0.69 to −0.39) and systolic blood pressure (r = −0.41; 95% confidence interval, −0.58 to −0.21). Single doses of 200 mg or more reduced 24 h ambulatory systolic and diastolic blood pressure by more than 10 mmHg and 5 mmHg, respectively, with effects maintained through 24 weeks. Part B evaluated the 800 mg dose under differing dietary salt conditions in 12 participants (zilebesiran, n = 8; placebo, n = 4) and showed attenuated blood pressure lowering with higher salt intake, consistent with RAAS inhibition. Part E evaluated combination therapy in 16 participants (zilebesiran alone, n = 6; zilebesiran plus irbesartan, n = 10) and suggested greater blood pressure lowering with the combination than with zilebesiran monotherapy [13].

### 5.2. Phase 2 Clinical Trials (KARDIA-1, KARDIA-2, and KARDIA-3)

The phase 2 KARDIA program extended evaluation of zilebesiran across monotherapy, add-on therapy, and higher-risk populations, providing a broader view of efficacy, safety, and potential clinical positioning.

KARDIA-1 enrolled 394 patients with mild-to-moderate hypertension after washout of prior antihypertensive therapy; 377 were included in the efficacy analysis [26]. Participants received zilebesiran 150, 300, or 600 mg every 6 months, zilebesiran 300 mg every 3 months, or placebo every 3 months. At month 3, all active regimens significantly reduced 24 h mean ambulatory systolic blood pressure versus placebo (least-squares mean difference, −14.1 to −16.7 mmHg; all *p* < 0.001). These effects were sustained through 6 months, particularly at doses of 300 mg or greater, supporting the feasibility of twice-yearly dosing [26].

KARDIA-2 evaluated zilebesiran as add-on therapy in patients whose hypertension remained uncontrolled despite indapamide, amlodipine, or olmesartan [35]. Indapamide is a thiazide-like diuretic that promotes natriuresis, amlodipine is a dihydropyridine calcium channel blocker that reduces vascular resistance, and olmesartan blocks the angiotensin II type 1 receptor. Diuretic and vasodilator mechanisms may complement upstream AGT suppression, whereas overlapping RAAS inhibition may partly explain the smaller effect with olmesartan. After a 4-week run-in period on background therapy, 663 of 1491 enrolled participants were randomized to a single 600 mg subcutaneous dose of zilebesiran or placebo. At 3 months, placebo-adjusted reductions in 24 h mean ambulatory systolic blood pressure were −12.1 mmHg with indapamide, −9.7 mmHg with amlodipine, and −4.5 mmHg with olmesartan [35].

KARDIA-3 (ClinicalTrials.gov identifier NCT06272487) evaluated zilebesiran in patients with uncontrolled hypertension and either established cardiovascular disease or high cardiovascular risk, with or without chronic kidney disease (CKD) [36,37]. In cohort A (estimated glomerular filtration rate [eGFR] of at least 45 mL/min/1.73 m^2^), 270 patients were randomized to a single 300 mg or 600 mg dose or placebo on background antihypertensive therapy [36]. At month 3, the 300 mg dose produced a placebo-adjusted reduction in office systolic blood pressure of 5.0 mmHg (*p* = 0.0431), whereas the 600 mg dose did not provide additional benefit (−3.3 mmHg; *p* = 0.1830). Because multiplicity adjustment required both doses to achieve *p* < 0.05 or one dose to achieve *p* < 0.025, the study did not meet its prespecified statistical criterion. Post hoc analyses suggested a stronger signal in patients receiving diuretic-containing regimens and in those with higher baseline systolic blood pressure. Cohort B (eGFR 30 to <45 mL/min/1.73 m^2^) later provided descriptive safety data; confirmed hyperkalemia and confirmed eGFR decline were infrequent, and no hypotension-related adverse events were observed [37]. Taken together, these data supported selection of zilebesiran 300 mg administered every 6 months for ZENITH, an ongoing phase 3 cardiovascular outcomes trial that is enrolling patients with uncontrolled hypertension and either established cardiovascular disease or high cardiovascular risk [38,39]. The KARDIA-3 efficacy and cohort B safety findings cited here derive from conference presentations rather than a full peer-reviewed trial publication and should therefore be interpreted as preliminary [36,37]. ZENITH trial design and recruitment status are based on a trial registry and sponsor materials [38,39]. No conclusion regarding reduction in cardiovascular events can currently be drawn from the observed blood pressure changes [36,37,38,39].

The design, efficacy, and selected safety findings of the major clinical trials are summarized in Table 1.

## 6. Clinical Safety and Tolerability

Assessing the safety profile of zilebesiran requires comparison across its own clinical program and, more cautiously, with other approved siRNA agents. Because these agents are used in different diseases, patient populations, and treatment settings, cross-trial comparisons should be interpreted descriptively rather than inferentially. Lumasiran is commonly associated with injection site reactions [40], givosiran with nausea, fatigue, renal dysfunction, transaminase elevations, and injection site reactions [41], and patisiran with infusion-related reactions [42]. Against this background, zilebesiran has shown a generally acceptable short-term safety profile across phase 1, KARDIA-1, KARDIA-2, and KARDIA-3. Among zilebesiran-treated participants in Parts A, B, and E of the phase 1 study, the most frequently reported adverse events were headache (19%), injection site reactions (6%), and upper respiratory tract infection (5%); no serious adverse event was considered treatment related [13]. In KARDIA-1, injection site reactions (6.3%) and hyperkalemia (5.3%) were the most frequent treatment-related events; no clinically meaningful deterioration in kidney function was observed [26]. In KARDIA-2, injection site reactions (3.0%), hypotension (4.3%), hyperkalemia (5.5%), and acute kidney injury or eGFR decline (4.9%) were reported more often with zilebesiran than placebo, but most events were mild, transient, and resolved without specific intervention; only 1.2% of patients required potassium binders [35]. KARDIA-3 cohort A likewise showed low rates of hypotension, hyperkalemia, and kidney dysfunction, while cohort B, which included patients with eGFR 30 to <45 mL/min/1.73 m^2^, showed low rates of confirmed hyperkalemia and confirmed eGFR decline and no reported hypotension adverse events [36,37].

RAAS inhibitors such as ACE inhibitors and ARBs remain central to the management of hypertension and cardiorenal disease [43,44,45], and they are also associated with class-specific adverse effects, including cough and angioedema [46], as well as hyperkalemia and worsening kidney function in selected patients [47]. In this context, the current zilebesiran evidence base is encouraging. A recent systematic review and meta-analysis reported no consistent clinically significant deterioration in serum creatinine or eGFR after zilebesiran exposure [48]. Overall, across studies with up to 6 months of follow-up, zilebesiran appears to be generally well tolerated, although larger and longer studies are still needed [26,35].

Selected adverse events are incorporated into Table 1. Across trials, safety findings have mainly involved injection site reactions, hypotension, hyperkalemia, and transient changes in kidney function; longer follow-up remains necessary.

## 7. Dosing Paradigm Shift: Redefining Chronic Hypertension Management

The receptor-mediated delivery platform of GalNAc–siRNA enables highly selective hepatocyte uptake and robust in vivo gene silencing [49,50], expanding the therapeutic potential of targeted oligonucleotides [51]. A defining feature of GalNAc–siRNA conjugates is prolonged pharmacodynamic durability, with target silencing that can persist for months after a single dose in preclinical and clinical settings [52]. This durability reflects several factors, including chemical modifications that increase nuclease resistance, efficient ASGPR-mediated uptake, and receptor recycling that sustains intracellular delivery [30,53].

Unlike conventional antihypertensive therapies that require daily oral administration and are often limited by poor adherence [54], zilebesiran may provide sustained control with infrequent dosing. Whether this pharmacologic convenience improves long-term adherence or outcomes in routine care remains unproven.

Emerging clinical data also support zilebesiran in combination regimens for heterogeneous forms of uncontrolled hypertension. In phase 1, coadministration with irbesartan appeared to augment blood pressure lowering without materially altering AGT suppression [13]. More systematic evidence came from KARDIA-2, in which add-on zilebesiran produced meaningful reductions in 24 h ambulatory systolic blood pressure when combined with indapamide or amlodipine, whereas the effect was smaller with olmesartan, possibly reflecting overlapping RAAS blockade [35]. KARDIA-3 extended evaluation to higher-risk patients receiving multidrug background therapy, and post hoc analyses suggested greater benefit on diuretic-containing regimens [36,55]. Together, these trials support durable blood pressure lowering and add-on efficacy across different background regimens, while the implications for treatment sequencing are considered in Section 11.

## 8. Personalized Dosing Strategies for Zilebesiran

Interindividual variability in response to zilebesiran raises the possibility of personalized dosing, although no randomized clinical trial has evaluated a biomarker-guided dose or dosing interval. Baseline serum AGT is an intuitive biomarker candidate because zilebesiran directly suppresses hepatic AGT synthesis [13,26]. Patients with higher circulating AGT levels could theoretically require different doses or dosing intervals, but AGT-guided treatment has not been formally studied. Genetic polymorphisms in the AGT gene have been associated with hypertension and other cardiometabolic phenotypes [56,57], but whether they influence the response to zilebesiran is unknown. Hepatic function or variation in ASGPR expression could theoretically modify drug uptake and pharmacodynamics, although these factors have not been evaluated as predictors of clinical response [13,30]. These concepts are mechanistically plausible and partly informed by preclinical biology, but they should not currently guide clinical dosing. Prospective studies would be required to determine whether biomarker- or phenotype-guided dosing has a practical role in hypertension care.

## 9. Comparison of Zilebesiran with Other Novel Antihypertensives

Persistent gaps in blood pressure control and medication adherence have accelerated development of mechanistically distinct antihypertensive therapies, including aldosterone synthase inhibitors (ASIs) [58], dual endothelin receptor antagonists [59], brain aminopeptidase A (AP-A) inhibitors [60], and RNA-based approaches [33]. Among these, zilebesiran is the most advanced nucleic acid therapy in clinical development for hypertension [61]. In early studies, it has shown durable 24 h blood pressure lowering with twice-yearly dosing and relatively low rates of serious adverse events [35].

Mechanistically, zilebesiran differs from these agents by suppressing hepatic AGT upstream of the entire RAAS [21]. By contrast, ASIs such as baxdrostat and lorundrostat are oral agents that inhibit cytochrome P450 family 11 subfamily B member 2 (CYP11B2) and reduce aldosterone synthesis [62]; aprocitentan is an oral dual endothelin receptor antagonist that blocks endothelin receptor type A (ETA) and endothelin receptor type B (ETB) signaling [63]; and firibastat inhibits brain AP-A, thereby reducing generation of central angiotensin III and downstream sympathetic and vasopressin-mediated effects [60]. Other RNA-based approaches, including antisense oligonucleotides, may also offer durability advantages, but targeted delivery and reversibility remain important challenges [33]. Because GalNAc-mediated delivery addresses hepatocyte targeting and supports sustained activity, zilebesiran occupies a distinctive position within this emerging therapeutic landscape.

**Figure 2:**
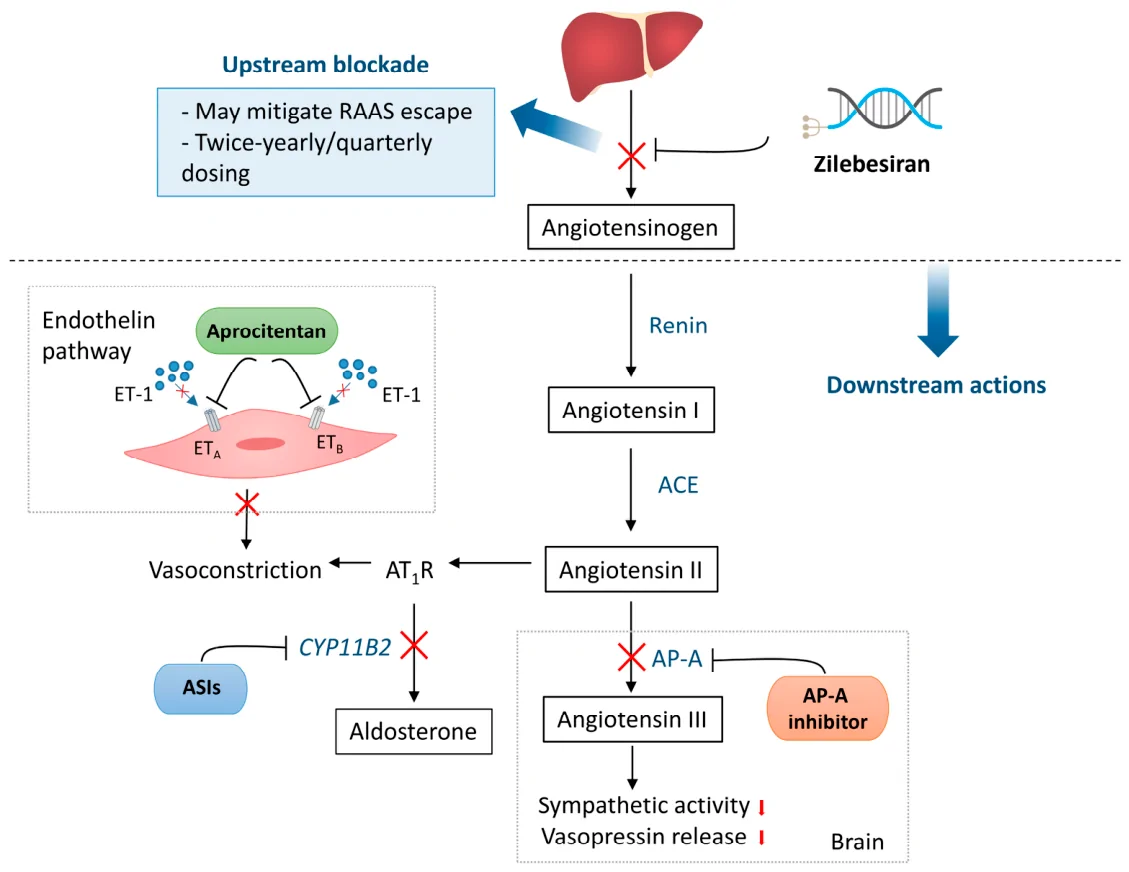
Mechanistic comparison of emerging antihypertensive classes. Zilebesiran suppresses hepatic AGT mRNA and provides upstream RAAS inhibition with infrequent subcutaneous dosing in early trials. ASIs reduce aldosterone synthesis, dual endothelin receptor antagonists block ETA and ETB receptors (shown by aprocitentan in the endothelin pathway box), and firibastat inhibits brain AP-A. ACE—angiotensin-converting enzyme; AGT—angiotensinogen; AP-A—aminopeptidase A; ASI—aldosterone synthase inhibitor; AT_1_R—angiotensin II type 1 receptor; CYP11B2—cytochrome P450 family 11 subfamily B member 2; ET-1—endothelin-1; ETA—endothelin receptor type A; ETB—endothelin receptor type B; mRNA—messenger RNA; RAAS—renin–angiotensin–aldosterone system.

Both baxdrostat and lorundrostat have produced clinically meaningful blood pressure reductions in uncontrolled or resistant hypertension [64,65,66,67,68], although they require daily oral dosing and ongoing biochemical monitoring [66]. Baxdrostat has progressed from encouraging early-phase data to positive phase 3 BaxHTN results, which showed significant placebo-adjusted systolic blood pressure reductions at 12 weeks [64,67,68]. In 2026, baxdrostat received approval in the United States as Baxfendy, the first approved aldosterone synthase inhibitor, for treatment of hypertension in combination with other antihypertensive medications in adults whose blood pressure is not adequately controlled [69]. Aprocitentan lowered 24 h ambulatory blood pressure in the phase 3 PRECISION study [70] and is already approved in the United States as add-on therapy for hypertension that is not adequately controlled on other drugs, although the risk of edema/fluid retention and pregnancy-related labeling restrictions remain important limitations [71]. Firibastat demonstrated mechanistic promise in early studies, but the phase 3 FRESH program did not show meaningful antihypertensive efficacy, so its current clinical role appears limited [60]. Figure 2 compares these emerging therapeutic classes. Table 2 provides a clinically oriented comparison of zilebesiran with established long-acting oral strategies and selected newer antihypertensive classes.

## 10. Zilebesiran in High-Risk Populations and Practical Monitoring

### 10.1. Chronic Kidney Disease

Early trials did not show clinically significant hyperkalemia or kidney function decline requiring medical intervention [13]. In KARDIA-2, mild hyperkalemia and transient eGFR decline were observed more often in zilebesiran-treated patients, but most abnormalities resolved on repeat measurement or with minimal intervention [35]. KARDIA-3 extended evaluation into CKD: cohort A included higher-risk patients with preserved or mildly reduced kidney function, whereas cohort B specifically enrolled patients with eGFR 30 to <45 mL/min/1.73 m^2^ [36,37]. In cohort B, most potassium elevations and eGFR declines were transient, confirmed abnormalities were infrequent, and no patient required hospitalization or dialysis because of hyperkalemia or kidney dysfunction [36,37]. If zilebesiran is approved for clinical use, serum creatinine and potassium would require careful monitoring, especially when it is combined with other agents that affect RAAS activity, volume status, or potassium handling.

### 10.2. Hepatic Impairment

Because zilebesiran depends on hepatocyte uptake, hepatic dysfunction could alter both delivery and pharmacodynamic response. No dedicated studies in clinically significant liver disease are available. In phase 1, a transient aspartate aminotransferase elevation was reported in one participant treated with 25 mg [13], and in KARDIA-1, seven participants had alanine aminotransferase or aspartate aminotransferase elevations that resolved spontaneously [26]. Until more data are available, caution is warranted in patients with clinically meaningful hepatic impairment.

### 10.3. Older Adults

Phase 1 included middle-aged participants with a mean age of 53.5 years (range 35 to 65 years) [13], whereas KARDIA-1 and KARDIA-2 expanded eligibility to older adults [26,35]. However, age-stratified analyses of blood pressure reduction, AGT suppression, and safety have not yet been reported in detail. Because older patients may have lower renin states, greater susceptibility to orthostatic symptoms, and more frequent kidney impairment, these factors would warrant cautious use and closer monitoring if zilebesiran is approved.

### 10.4. Diabetes and Established Cardiovascular Disease

The earliest zilebesiran studies excluded patients with diabetes and those with prior major cardiovascular events, limiting generalizability to populations at highest absolute risk. KARDIA-3 partly addressed this gap by enrolling patients with established cardiovascular disease or high cardiovascular risk [36,37]. Nevertheless, the long-term safety and effectiveness of sustained AGT suppression in patients with diabetes, heart failure, and advanced vascular disease remain uncertain and require dedicated study.

### 10.5. Pregnancy

Preclinical work in rat models of preeclampsia did not show placental transfer or obvious harm to offspring [72], but these data are insufficient to establish safety in human pregnancy. Given the known fetal toxicity of conventional RAAS blockade, zilebesiran should currently be regarded with caution in pregnancy until dedicated reproductive safety data become available.

## 11. Potential Clinical Positioning and Patient Selection if Approved

If approved, zilebesiran would most likely be integrated with established first-line agents rather than replace them. KARDIA-2 provides randomized evidence that coadministration with indapamide or amlodipine can produce additive blood pressure lowering, with a smaller effect observed on an olmesartan background [35]. Potential candidates could include patients with persistently poor adherence to daily medication, resistant hypertension after confirmation of adherence and exclusion of secondary causes, high pill burden, or a need for a simplified treatment schedule. Patients whose blood pressure remains uncontrolled despite combination therapy might also be considered, particularly when a diuretic-containing regimen is tolerated. These scenarios represent hypothetical clinical positioning rather than validated indications, and no trial has yet shown that zilebesiran improves adherence, reduces pill burden in practice, or is superior to optimized conventional therapy. Any future use would require careful assessment of baseline kidney function and potassium, the ability to attend follow-up despite infrequent dosing, and the risk of hypotension during acute illness. Practical implementation would also require training in subcutaneous administration, attention to injection site rotation and dosing interval adherence, and monitoring of potassium and creatinine, particularly during illness, dehydration, or intercurrent changes in background therapy.

## 12. Translational Challenges and Unforeseen Consequences

Clinical implementation of zilebesiran raises questions that differ from those associated with conventional antihypertensive drugs. A reduction of more than 90% in circulating AGT may substantially alter tissue and systemic RAAS signaling, because angiotensin generation in several organs may depend, to varying degrees, on hepatic AGT supply [22]. Marked AGT depletion triggers compensatory renin elevation and can effectively collapse downstream Ang II generation, which may be desirable for blood pressure control but could impair physiological responses needed to maintain hemodynamic stability [33]. These concerns are most relevant during septic shock, hemorrhage, severe hypovolemia, or acute kidney injury, circumstances in which endogenous RAAS activation becomes protective [16,73]. As with other forms of intense RAAS blockade, on-target toxicity could include hypotension, hyperkalemia, and acute kidney injury [74]. Pregnancy is another concern, because RAAS signaling contributes to maternal cardiovascular adaptation and fetal development [33,34].

Another challenge is external validity. Most current clinical data derive predominantly from White participants in high-income countries [13,26], which limits confidence about generalizability to regions bearing the greatest global burden of hypertension. Larger and more diverse studies are needed, particularly in patients with diabetes, CKD, heart failure, and established cardiovascular disease [33]. KARDIA-3 was designed as an initial step toward higher-risk populations [55], but broader representation remains essential.

These concerns have stimulated interest in strategies capable of restoring RAAS activity or supporting blood pressure in emergencies. The available reversal evidence remains preclinical and is summarized below [75,76].

## 13. Reversal Strategies for siRNA-Mediated Blood Pressure Reduction

The clinical viability of AGT-targeting siRNA will depend in part on whether its antihypertensive effects can be mitigated quickly when needed. In a preclinical study, spontaneously hypertensive rats received AGT-siRNA (10 mg/kg every 2 weeks) for 4 weeks while on a low-salt diet. During the final 2 weeks, animals were randomized to fludrocortisone, midodrine, or a high-salt diet, and pressor responses to norepinephrine and Ang II were assessed [76]. siRNA therapy depleted AGT by more than 99% and lowered mean arterial pressure by 19 mmHg. Exogenous Ang II and norepinephrine restored mean arterial pressure rapidly, whereas fludrocortisone and a high-salt diet produced more gradual improvement; midodrine had little effect [76].

REVERSIR, an oligonucleotide complementary to the antisense strand of the siRNA, represents a more targeted reversal concept. In preclinical models, REVERSIR is taken up by hepatocytes, binds RISC-associated siRNA, terminates gene silencing, and restores target protein levels within several days [75,77]. This approach has not been tested as a reversal strategy in patients receiving zilebesiran and therefore remains experimental.

No clinical protocol has been validated specifically for reversal of zilebesiran. If a patient treated with zilebesiran developed acute hemodynamic compromise, standard supportive care, including intravascular volume resuscitation and conventional vasopressors such as norepinephrine, would remain the clinically established foundation. Exogenous Ang II could theoretically bypass upstream AGT suppression, but this concept is supported by mechanistic reasoning and preclinical findings rather than human zilebesiran reversal data [73,76]. Close monitoring of serum potassium and kidney function would also be appropriate because hyperkalemia and acute kidney injury may accompany profound RAAS inhibition.

Accordingly, the reversal approaches discussed above should be viewed as preclinical or theoretical. No nucleic acid-based reversal strategy has entered routine clinical use, and efficacy, timing, availability, and safety in humans remain to be established [75,76,77].

## 14. Future Directions

Zilebesiran has expanded interest in RNA-based antihypertensive therapy, but several research priorities remain. Prospective studies are needed to determine whether the candidate biomarkers discussed in Section 8 can support individualized dosing. Combination strategies also require further evaluation [13,35,36]. Whether stable blood pressure control could permit a safe reduction in daily pill burden remains unknown.

Large, diverse, long-term studies are also needed to define safety in CKD, diabetes, heart failure, and other high-risk settings. Large-scale adoption will additionally depend on efficient oligonucleotide synthesis, reproducible GalNAc conjugation, stringent purification and quality control, manufacturing capacity, and cost; these requirements may limit access in resource-constrained settings [33]. Regulatory and health-economic evaluation will therefore be central to equitable implementation.

## 15. Limitations

This review has several limitations. The evidence base remains dominated by early-phase studies with modest sample sizes, relatively short follow-up, and limited demographic diversity. Long-term cardiovascular and renal outcomes are unavailable, and expected cardiovascular benefit remains speculative pending completion of ZENITH [38,39]. KARDIA-3 results and ZENITH status information derive from conference, registry, or sponsor sources rather than full peer-reviewed reports and therefore require cautious interpretation [36,37,38,39]. Finally, personalized dosing and the proposed patient-selection scenarios remain unvalidated, while emergency reversal strategies are supported only by preclinical evidence [75,76,77].

## 16. Conclusions

Zilebesiran represents an important advance in antihypertensive drug development. By using RNAi to suppress hepatic AGT, it addresses two persistent limitations of conventional care: the need for frequent dosing and the potential for RAAS escape. Early clinical trials show potent, dose-dependent, and durable reductions in serum AGT and blood pressure after a single subcutaneous dose, with the strongest data supporting dosing every 6 months. Add-on studies also demonstrate additional blood pressure lowering with selected conventional agents, particularly diuretics and calcium channel blockers. These conclusions concern blood pressure lowering and should not be extrapolated to cardiovascular event reduction [13,26,35,36,37].

At the same time, enthusiasm should be tempered by the need for longer follow-up and broader evidence. The short-term safety profile appears acceptable, but the consequences of sustained, profound AGT suppression in diverse high-risk populations remain uncertain. Reliable reversal strategies would be important if this therapy entered routine practice, but current targeted reversal concepts remain preclinical. The ongoing ZENITH phase 3 trial is testing whether the durable blood pressure lowering achieved with zilebesiran translates into meaningful reductions in cardiovascular events [38]. Until ZENITH reports cardiovascular outcome data, any expected benefit on major cardiovascular events remains speculative, and zilebesiran should be regarded as an investigational therapy.

## Figures and Tables

**Table 1:** Design, efficacy, and selected safety findings of clinical trials evaluating zilebesiran.

Study/Phase	Location	Sample Size	Dosing Regimen	Main BP Outcome (Ambulatory Unless Stated)	Selected Safety Findings/Status
**Phase** **1**	United Kingdom; 4 sites	107 enrolled	Single SC dose, 10–800 mg	At doses ≥200 mg, reductions >10 mmHg at week 8, sustained through week 24 [13]	Mostly mild injection site reactions; no treatment-related serious adverse events [13]
**KARDIA-1** **(phase 2)**	Canada, Ukraine, United Kingdom, and United States; 78 sites	394 randomized; 377 analyzed	150, 300, or 600 mg every 6 months; 300 mg every 3 months	Placebo-adjusted change at month 3: −14.1 to −16.7 mmHg [26]	Hypotension 4.3%; hyperkalemia 5.3%; renal adverse events 1.3% [26]
**KARDIA-2** **(phase 2)**	Canada, Estonia, Germany, Latvia, Lithuania, Poland, United Kingdom, and United States; 150 sites	1491 enrolled; 663 randomized	Single 600 mg dose added to indapamide, amlodipine, or olmesartan	Placebo-adjusted change at month 3: −12.1, −9.7, and −4.5 mmHg, respectively [35]	Hypotension 4.3%; hyperkalemia 5.5%; acute kidney failure/eGFR decline 4.9% [35]
**KARDIA-3** **(phase 2)**	Australia, Canada, Puerto Rico, Switzerland, United Kingdom, and United States; 195 sites	375 participants; cohort A, 270 randomized	300 or 600 mg (cohort A); 150, 300, or 600 mg (cohort B)	300 mg: placebo-adjusted office SBP −5.0 mmHg at month 3; multiplicity criterion not met [36,37]	Conference-reported evidence; infrequent hyperkalemia and eGFR decline in cohort B [36,37]
**ZENITH** **(phase 3)**	Global	Target ≈ 11,000	300 mg every 6 months plus standard care	No results available; cardiovascular outcomes trial ongoing	Ongoing; no cardiovascular outcome data available [38,39]

BP—blood pressure; eGFR—estimated glomerular filtration rate; SBP—systolic blood pressure; SC—subcutaneous.

**Table 2:** Clinical comparison of zilebesiran with established long-acting oral strategies and selected newer antihypertensive therapies.

Strategy/Agent	Evidence and Regulatory Status	Limitations and Monitoring	Potential Advantages	Mechanism and Dosing
Established once-daily oral agents and single-pill combinations	Established randomized and outcomes evidence; widely approved [43,44,45,46,47,54]	Daily adherence remains necessary; class-specific adverse effects; single-pill combinations may not eliminate pill burden	Extensive clinical experience; flexible titration; cardiovascular outcome evidence for established classes; low-cost options	Established RAAS blockers, calcium channel blockers, and diuretics; generally once daily
Zilebesiran	Phase 1 and randomized phase 2 evidence for blood pressure lowering and short-term safety; cardiovascular outcomes unproven; investigational, with ZENITH ongoing [13,26,35,36,37,38,39]	Limited reversibility and titratability; monitoring for hypotension, hyperkalemia, and kidney dysfunction; long-term safety and adherence effects unknown	Durable 24 h blood pressure lowering; infrequent dosing; add-on effect with selected background therapies	Hepatic AGT mRNA silencing; subcutaneous administration every 6 months in current trials
Baxdrostat	Randomized phase 3 evidence; FDA-approved in 2026 for use in combination with other antihypertensive drugs [64,67,68,69]	Hyperkalemia and hyponatremia; potassium and sodium monitoring; daily adherence required	Effective in uncontrolled or resistant hypertension; complementary aldosterone-targeted mechanism	Selective aldosterone synthase inhibition; oral once daily
Lorundrostat	Randomized phase 2 and phase 3 evidence; investigational in the sources reviewed [58,62,65,66]	Hyperkalemia and kidney function monitoring; daily adherence required	Blood pressure lowering in uncontrolled and resistant hypertension	Selective aldosterone synthase inhibition; oral once daily
Aprocitentan	Randomized phase 3 evidence; approved in the United States as add-on therapy [59,63,70,71]	Edema/fluid retention, hepatotoxicity, decreased hemoglobin, and embryo-fetal toxicity; liver function monitoring and pregnancy testing; daily adherence required	Evidence in resistant hypertension; complementary non-RAAS mechanism	Dual ETA/ETB receptor antagonism; oral once daily
Firibastat	Negative phase 3 efficacy; not approved for hypertension [60]	Phase 3 program did not demonstrate meaningful efficacy; twice-daily adherence	Mechanistically distinct central pathway, but no demonstrated clinical advantage	Brain AP-A inhibition; oral twice daily

AGT—angiotensinogen; AP-A—aminopeptidase A; ETA—endothelin receptor type A; ETB—endothelin receptor type B; FDA—U.S. Food and Drug Administration; mRNA—messenger RNA; RAAS—renin–angiotensin–aldosterone system. Regulatory status is reported as described in the sources available through May 2026; zilebesiran has no approved indication.

## Data Availability

No new data were created or analyzed in this study. Data sharing is not applicable to this article.
